# Electrophysiological Properties of Melanin-Concentrating Hormone and Orexin Neurons in Adolescent Rats

**DOI:** 10.3389/fncel.2018.00070

**Published:** 2018-03-13

**Authors:** Victoria Linehan, Michiru Hirasawa

**Affiliations:** Division of Biomedical Sciences, Faculty of Medicine, Memorial University, St. John’s, NL, Canada

**Keywords:** melanin-concentrating hormone, orexin, hypocretin, lateral hypothalamus, development, adolescence, patch clamp, short-term synaptic plasticity

## Abstract

Orexin and melanin-concentrating hormone (MCH) neurons have complementary roles in various physiological functions including energy balance and the sleep/wake cycle. *in vitro* electrophysiological studies investigating these cells typically use post-weaning rodents, corresponding to adolescence. However, it is unclear whether these neurons are functionally mature at this period and whether these studies can be generalized to adult cells. Therefore, we examined the electrophysiological properties of orexin and MCH neurons in brain slices from post-weaning rats and found that MCH neurons undergo an age-dependent reduction in excitability, but not orexin neurons. Specifically, MCH neurons displayed an age-dependent hyperpolarization of the resting membrane potential (RMP), depolarizing shift of the threshold, and decrease in excitatory transmission, which reach the adult level by 7 weeks of age. In contrast, basic properties of orexin neurons were stable from 4 weeks to 14 weeks of age. Furthermore, a robust short-term facilitation of excitatory synapses was found in MCH neurons, which showed age-dependent changes during the post-weaning period. On the other hand, a strong short-term depression was observed in orexin neurons, which was similar throughout the same period. These differences in synaptic responses and age dependence likely differentially affect the network activity within the lateral hypothalamus where these cells co-exist. In summary, our study suggests that orexin neurons are electrophysiologically mature before adolescence whereas MCH neurons continue to develop until late adolescence. These changes in MCH neurons may contribute to growth spurts or consolidation of adult sleep patterns associated with adolescence. Furthermore, these results highlight the importance of considering the age of animals in studies involving MCH neurons.

## Introduction

Neurons expressing melanin-concentrating hormone (MCH) and orexin (also known as hypocretin) comprise distinct cell populations co-localized within the lateral hypothalamus, and have complementary roles in several physiological functions including food intake, metabolism, motivation and sleep/wake cycle (Barson et al., 2013). To investigate the function of these neurons at the cellular level, *in vitro* electrophysiological studies commonly use rodents in the early post-weaning period when cells are thought to be less vulnerable to the mechanical damage caused by brain slice procedures. However, the post-weaning period in rodents corresponds to adolescence, a period of ongoing neural, physiological and behavioral maturation (Steinberg, 2005). At the cellular level, adolescence is associated with various neuronal changes including neuron number (Markham et al., 2007), synaptic connections and dendritic structure (McKay and Turner, 2005; Groen et al., 2014). Functional neuronal changes also occur during this period, affecting intrinsic excitability, synaptic transmission and plasticity (McKay and Turner, 2005; Kasanetz and Manzoni, 2009; Groen et al., 2014). Therefore, it is possible that orexin and MCH neurons are not fully mature by weaning. If so, findings in post-weaning, adolescent animals may not always be applicable to mature neurons in adults. Furthermore, a delayed maturation of orexin and MCH neurons could have functional implications in growth spurts, since these neurons play essential roles in energy homeostasis (Barson et al., 2013). In support of this idea, MCH deficiency results in a reduction in body length and weight in adolescent rats (Mul et al., 2010). In addition, as both orexin and MCH neurons are involved in regulation of the sleep/wake cycle (Modirrousta et al., 2005; Hassani et al., 2009), maturation of these neurons could also contribute to the consolidation of adult sleep rhythms, which coincides with pubertal changes in rats (Hagenauer and Lee, 2013). These neurons may also affect sexual maturation through regulation of gonadotropin-releasing hormone (GnRH) neurons (Wu et al., 2009; Gaskins and Moenter, 2012) and gonadotropin release (Murray et al., 2000; Tsukamura et al., 2000; Small et al., 2003).

There are previous studies that described age-dependent changes in the electrophysiological properties of orexin and MCH neurons during the postnatal period (Li and van den Pol, 2009; Ogawa et al., 2017). However, no study has focused on the changes during the post-weaning period. In the present study, we characterized the electrophysiological properties of orexin and MCH neurons in rats at several time points post-weaning. Our results show that MCH neurons undergo an age-dependent reduction in excitability during adolescence, but not orexin neurons. These results suggest the importance of carefully considering the age in studies involving these neurons and the potential developmental changes in their functional output.

## Materials and Methods

This study was carried out in accordance with the recommendations of the Canadian Council on Animal Care. The protocol was approved by Memorial University’s Institutional Animal Care Committee. Male Sprague-Dawley rats were at postnatal day 21 (Charles River, Senneville, QC, Canada) upon arrival, were housed in light and temperature-controlled room (12 h light: 12 h dark, lights on at 7:00 AM) and had *ad libitum* access to standard chow (Prolab RMH 3000) and water.

A total of 308 cells were studied from 94 rats. Rats were sacrificed by decapitation under deep isoflurane anesthesia at 4, 7 and 14 weeks of age at 9:00–9:30 AM. Coronal slices of the hypothalamus (250 μm) were generated using a vibratome (VT-1000, Leica Microsystems) in cold artificial cerebral spinal fluid (ACSF; in mM: 126 NaCl, 2.5 KCl, 1.2 NaH_2_PO_4_, 1.2 MgCl_2_, 18 NaHCO_3_, 2.5 glucose, and 2 CaCl_2_) bubbled with 95% O_2_/5% CO_2._ Slices were incubated in ACSF at 32–34°C for 30 min then at room temperature until recording. Hemisected slices were transferred into a recording chamber and perfused with ACSF (30–32°C), visualized with infrared-differential interference contrast optics (DM LFSA, Leica Microsystems), and cells in the lateral hypothalamus/perifornical area and zona incerta were selected for study. No region-specific differences in electrophysiological parameters were found in identified MCH and orexin neurons within these regions, thus data were combined. A glass pipette was filled with an internal solution (in mM: 123 K-gluconate, 2 MgCl_2_, 1 KCl, 0.2 EGTA, 10 HEPES, 5 Na_2_ATP, 0.3 NaGTP, and 2.7 biocytin; tip resistance 3–5 MΩ). Whole cell patch clamp recordings were performed using pClamp 9/10 software and Multiclamp 700B (Molecular Devices) with series/access resistance of 10–25 MΩ. For voltage clamp experiments, the holding potential was −70 mV, and 50-ms, 20-mV square pulse was applied every 60 s to monitor series/access resistance. Signals were filtered at 1 kHz and digitized at 5–10 kHz. Voltage measurements were corrected for liquid junction potential (−14.9 mV).

Once whole cell access was achieved, a series of 600 ms hyperpolarizing and depolarizing current injections were applied to cells in 50 pA increments. Unique electrophysiological responses to these current injections were used to identify MCH and orexin neurons as described previously (Linehan et al., 2015; Belanger-Willoughby et al., 2016) and to assess active membrane properties. Briefly, MCH neurons are relatively hyperpolarized and silent at rest, and lack H-current. Positive current injections often induce A-like current and spike accommodation. Orexin neurons are typically spontaneously active, show H-current and rebound depolarization during and after hyperpolarizing current injections, respectively. Both cell types have uniphasic afterhyperpolarizing potentials (AHP).

**Figure 1:**
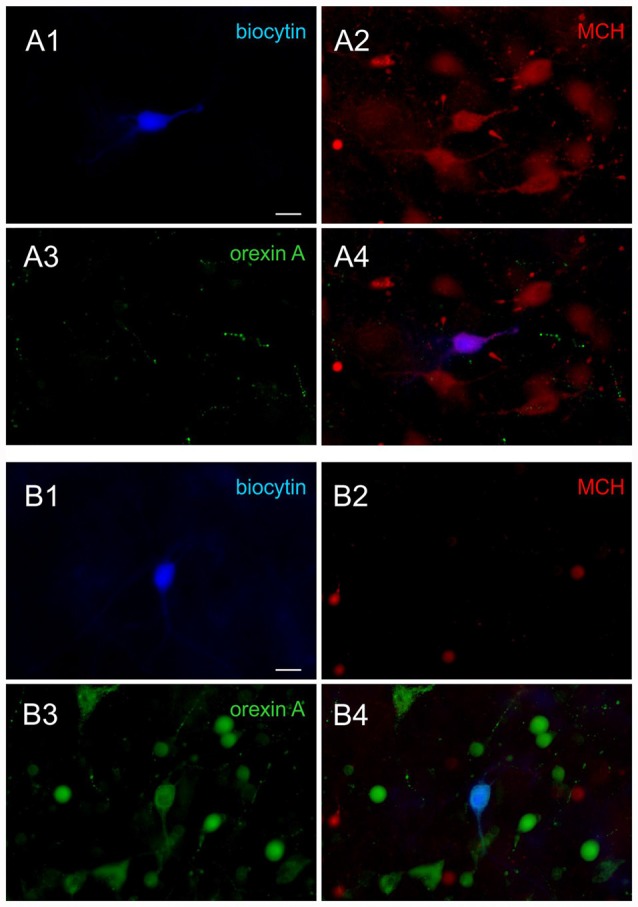
*Post hoc* immunohistochemical identification of melanin-concentrating hormone (MCH) and orexin neurons. **(A)** Sample images of a confirmed MCH neuron. *Post hoc* immunohistochemical staining of the biocytin labeled cell (**A1**, blue) shows co-localization with MCH (**A2**, red) but not orexin A (**A3**, green), shown by overlap **(A4)**. **(B)** Sample images of a confirmed orexin neuron. The biocytin stained cell **(B1)** is co-localized with orexin A **(B3)** but not MCH **(B2)**, shown by overlap **(B4)**. Scale bar: 20 μm.

Following the recording, the neurochemical phenotype was confirmed in a subset of recorded neurons filled with biocytin using triple staining for biocytin, MCH and orexin A, as previously described (Linehan et al., 2015; Belanger-Willoughby et al., 2016). Briefly, brain slices were fixed in 10% formalin overnight, and then incubated in a cocktail of rabbit anti-MCH IgG (1:2000; H-070-47, Phoenix Pharmaceuticals, Burlingame, CA, USA) and goat anti-orexin A IgG (1:2000; sc8070, Santa Cruz Biotechnology Inc., Dallas, TX, USA) for 3 days. This was followed by appropriate secondary antibodies and Alexa 350-conjugated streptavidin (1:500) to visualize biocytin (Figure 1). All neurons that were subjected to *post hoc* immunohistochemistry had matching electrophysiological properties and neurochemical phenotype (90 of 90 MCH neurons, 100 of 100 orexin neurons, total 190 cells confirmed; 100% accuracy).

To evaluate cell excitability, the resting membrane potential (RMP) and the response to a series of 600 ms depolarizing current injections were analyzed. RMP was measured immediately prior to current injection when the cell was not firing. The first spike latency was the time from the onset of current injection to first action potential (AP). In some MCH neurons, +50 and 100 pA injections did not elicit firing, in which case the duration of the current injection (600 ms) was assigned for first spike latency. In contrast, 150 and 200 pA elicited firing in every MCH neuron, while all orexin neurons fired during 50–200 pA current injections. The following definitions were used for AP waveform analysis: AP threshold was the membrane potential where the slope of the trace equaled 10 mV/ms; AP amplitude was the baseline to peak; AP peak was the membrane potential where AP peaked; half-width was the duration of AP where its amplitude was at 50%; 10%–90% decay was the time it took to decay from 10% to 90% of AP peak to baseline; and AHP amplitude was measured from the baseline to the peak of AHP.

To study evoked EPSCs, a glass electrode filled with ACSF was placed adjacent to the recorded cell to stimulate afferent fibers. The chloride channel blocker picrotoxin (50 μM, Sigma-Aldrich Canada, Oakville, ON, Canada) was bath applied to isolate glutamatergic excitatory postsynaptic currents (EPSCs) and the voltage-gated Na^+^ channel blocker tetrodotoxin (TTX, 1 μM, Alomone Labs, Jerusalem) was used to isolate miniature EPSC (mEPSC). Train stimulation protocols consisted of 60 pulses at 10 Hz or 50 pulses at 50 Hz, every 60 s, and the amplitude of EPSCs elicited by each stimulation was measured. The paired-pulse ratio (PPR) was calculated as the amplitude of the second EPSC divided by the first EPSC (EPSC2/EPSC1). All EPSCs during train stimulation were normalized to the amplitude of the first EPSC. The initial synaptic facilitation was the average of 2nd to 5th EPSCs, while the plateau was the average of the last five EPSCs.

Data are expressed as mean ± SEM. The number of samples is reported as *n*/*N* where *n* indicates the number of cells and *N* is the number of animals. Evoked EPSC amplitude and AP waveform parameters were analyzed using pClamp 10 (Molecular Devices). mEPSCs were analyzed by Minianalysis software (Synaptosoft Inc., Leonia, NJ, USA). For MCH neurons, 81–505 mEPSCs were analyzed per cell. For orexin neurons, 168–2313 mEPSCs were analyzed per cell. Statistical tests were performed using Prism 6 (GraphPad Software Inc., San Diego, CA, USA), including paired *t*-tests and one- or two-way analysis of variance (ANOVA) and one- or two-way repeated measures (RM) ANOVA. When significance was found with ANOVA, Holm-Sidak multiple comparison tests were performed and the results are indicated in figures. *p* < 0.05 was considered significant.

## Results

The excitability of MCH and orexin neurons were examined at early adolescence (4 weeks old), late adolescence (7 weeks old), and adulthood (14 weeks old; Spear, 2000; Sengupta, 2013).

### Intrinsic Excitability of MCH Neurons During the Post-Weaning Period

**Figure 2:**
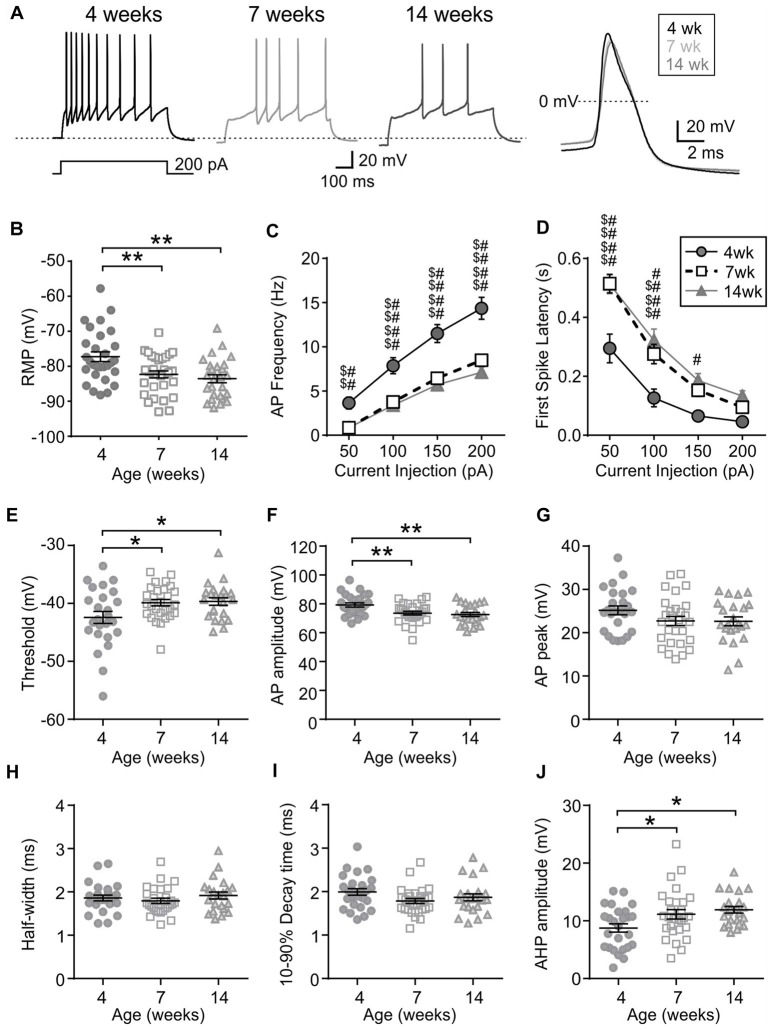
MCH neurons undergo an age-dependent decrease in excitability. **(A)** Sample traces during a 200-pA current injection (left) and averaged action potential (AP) waveform (right) recorded from MCH neurons of 4, 7 and 14-week old rats. Dotted reference line in the left panel is −80 mV. **(B)** Resting membrane potential (RMP) of MCH neurons from different age groups.** (C,D)** AP frequency **(C)** and first spike latency **(D)** of MCH neurons during positive current injections. **(E–J)** Various parameters of AP waveform as indicated on the Y-axis. *p < 0.05, **p < 0.01, one-way analysis of variance (ANOVA) with Holm–Sidak post-test. 4 weeks vs. 7 weeks: ^$$^*p* < 0.01, ^$$$^*p* < 0.001, ^$$$$^*p* < 0.0001; 4 weeks vs. 14 weeks: ^#^*p* < 0.05, ^##^*p* < 0.01, ^####^*p* < 0.0001, two-way repeated measures (RM) ANOVA with Holm-Sidak post-test.

First, the intrinsic excitability of MCH neurons was examined. Compared to those from 4-week old rats, MCH neurons of 7- and 14-week old rats had a hyperpolarized RMP (4 weeks *n*/*N* = 29/21, 7 weeks *n*/*N* = 31/13 and 14 weeks *n*/*N* = 26/9; *p* = 0.0008, one-way ANOVA; Figures 2A,B). During positive current injections of varying amplitude, MCH neurons from 4-week old rats responded with higher AP frequency (*p* < 0.0001, two-way RM ANOVA; Figure 2C) and a shorter latency to first spike compared to 7- and 14-week olds (*p* < 0.0001, two-way RM ANOVA; Figure 2D). Furthermore, analysis of AP waveform revealed that the threshold was significantly lower (*p* = 0.0231, one-way ANOVA; Figure 2E) and AP amplitude was larger at 4 weeks compared to those at older age (*p* = 0.0020, one-way ANOVA; Figure 2F), but without any difference in the absolute AP peak (*p* = 0.1532, one-way ANOVA; Figure 2G). The half-width (*p* = 0.4429, one-way ANOVA; Figure 2H) and decay time also did not change with age (*p* = 0.1094, one-way ANOVA; Figure 2I), whereas the amplitude of AHP became larger between 4 weeks and 7 weeks of age (*p* = 0.0093, one-way ANOVA; Figure 2J). Overall these results suggest that the excitability of MCH neurons decreases during adolescence and reaches the adult level by 7 weeks of age.

### Age-Dependent Changes in Excitatory Transmission to MCH Neurons

**Figure 3:**
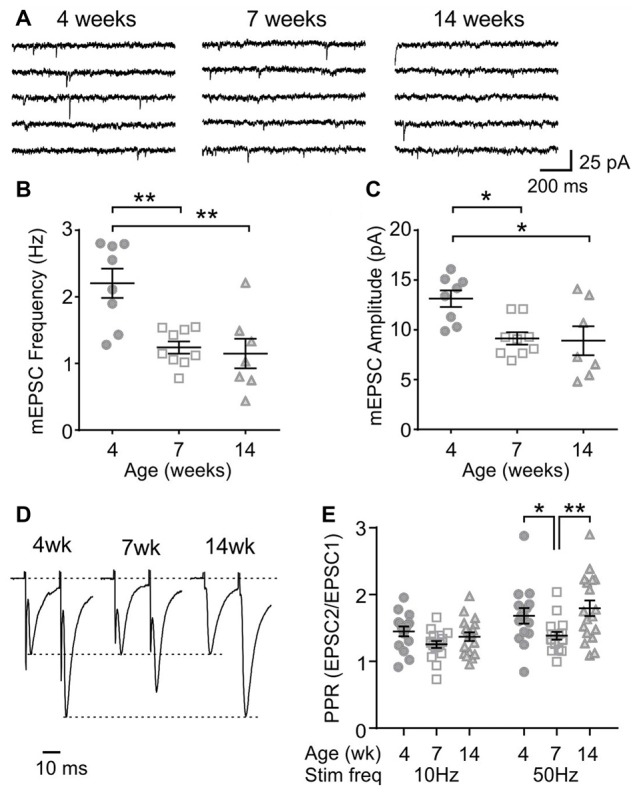
Age-dependent changes in excitatory synaptic transmission to MCH neurons. **(A)** Sample traces of miniature excitatory postsynaptic currents (mEPSCs) recorded from MCH neurons of 4, 7 and 14-week old rats. **(B,C)** Frequency **(B)** and amplitude **(C)** of mEPSCs in MCH neurons. **(D)** Pairs of EPSCs evoked at 50 Hz recorded from MCH neurons. Traces are scaled to the first EPSC. **(E)** Paired pulse ratio (PPR) at 10 and 50 Hz. *p < 0.05, **p < 0.01, one-way ANOVA **(B,C)** or two-way RM ANOVA **(E)** with Holm-Sidak post-test.

Next, to determine any age-dependent change in excitatory synaptic transmission, mEPSCs were examined. In MCH neurons, the frequency and amplitude of mEPSC was lower at 7 and 14 weeks compared to those at 4 weeks (4 weeks *n*/*N* = 8/5, 7 weeks *n*/*N* = 9/9 and 14 weeks *n*/*N* = 7/5; mEPSC frequency *p* = 0.0007, mEPSC amplitude *p* = 0.0082, one-way ANOVA; Figures 3A–C), suggesting an ongoing maturation of excitatory synaptic connections during early adolescence. The PPR, a measure of presynaptic release probability, also changed significantly over the period examined when paired pulses were applied at 50 Hz (4 weeks *n*/*N* = 15/8, 7 weeks *n*/*N* = 17/10 and 14 weeks *n*/*N* = 18/10; *p* = 0.0156, two-way RM ANOVA; Figures 3D,E). Specifically, PPR was lower at 7 weeks compared to 4 or 14 weeks of age.

**Figure 4:**
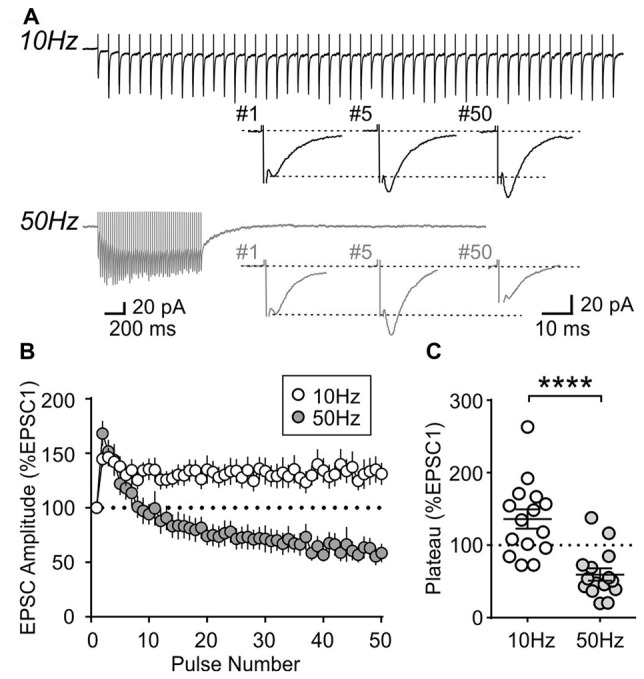
Activity-dependent short-term plasticity in MCH neurons of 4-week old rats. **(A)** Sample recordings of evoked EPSCs during 10 and 50 Hz trains. Expanded traces below show the 1st, 5th and 50th EPSC of the train. **(B)** Normalized amplitude of 50 EPSCs during 10 and 50 Hz train stimulation. **(C)** Plateau of EPSC amplitude during 10 and 50 Hz train stimulation. ****p < 0.0001, paired *t*-test.

To further characterize the synaptic properties of MCH neurons, train stimulation was applied to excitatory afferents (Figure 4A). During a 10 Hz train, most MCH neurons tested from 4-week old rats showed a sustained synaptic facilitation. On the other hand, 50 Hz stimulation induced a brief synaptic facilitation, followed by a steady synaptic depression (*n*/*N* = 15/8; 10 vs. 50 Hz, *p* < 0.0001, paired *t*-test; Figures 4B,C). These results suggest that short-term plasticity in MCH neurons can be either facilitating or depressing depending on the frequency and duration of stimulation.

**Figure 5:**
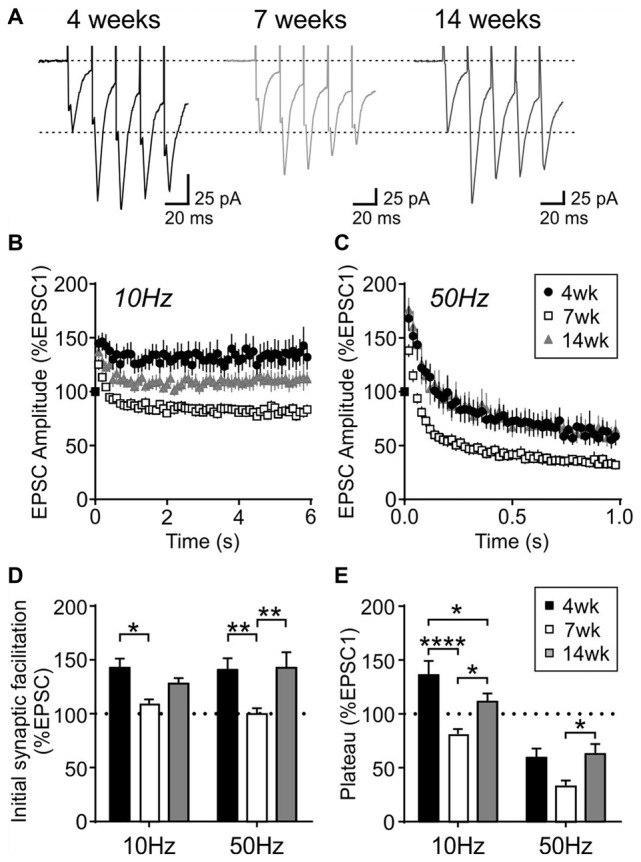
Activity-dependent short-term plasticity in MCH neurons is modulated by age. **(A)** Sample traces of EPSCs evoked at 50 Hz in MCH neurons (first five EPSCs are shown). **(B,C)** Normalized EPSC amplitude during 10 Hz **(B)** and 50 Hz trains **(C)** from different age groups as indicated. Note that the 4-week data is also shown in Figure 4B. **(D)** Synaptic facilitation at the beginning of 10 Hz and 50 Hz trains (average of EPSC2–5, normalized to EPSC1). **(E)** EPSC plateau at the end of 10 Hz and 50 Hz trains (average of EPSC26–30, normalized to EPSC1). *p < 0.05, **p < 0.01, ****p < 0.0001, two-way RM ANOVA with Holm-Sidak post-test.

Interestingly, this short-term plasticity was also affected by age. The sustained synaptic facilitation induced by 10 Hz train observed in the 4-week old group was no longer observed at 7 weeks (*n*/*N* = 17/10) or 14 weeks (*n*/*N* = 18/10). At 7 weeks, a brief synaptic facilitation was followed by a synaptic depression, whereas at 14 weeks there was no synaptic depression; instead the synaptic strength completely returned to the baseline level following a brief synaptic facilitation (Figures 5B,D,E). Age-dependent changes were also seen with 50 Hz stimulation: at 7 weeks, the initial facilitation was smaller (*p* = 0.0024, two-way RM ANOVA) and the steady-state synaptic depression (plateau) was more prominent compared to 4- and 14-week old groups (*p* = 0.0015, two-way RM ANOVA; Figures 5A,C–E). These results suggest that excitatory synapses to MCH neurons have higher release probability and are more prone to synaptic fatigue in 7-week old relative to 4- and 14-week old rats.

### Electrophysiological Properties of Orexin Neurons During the Post-Weaning Period

**Figure 6:**
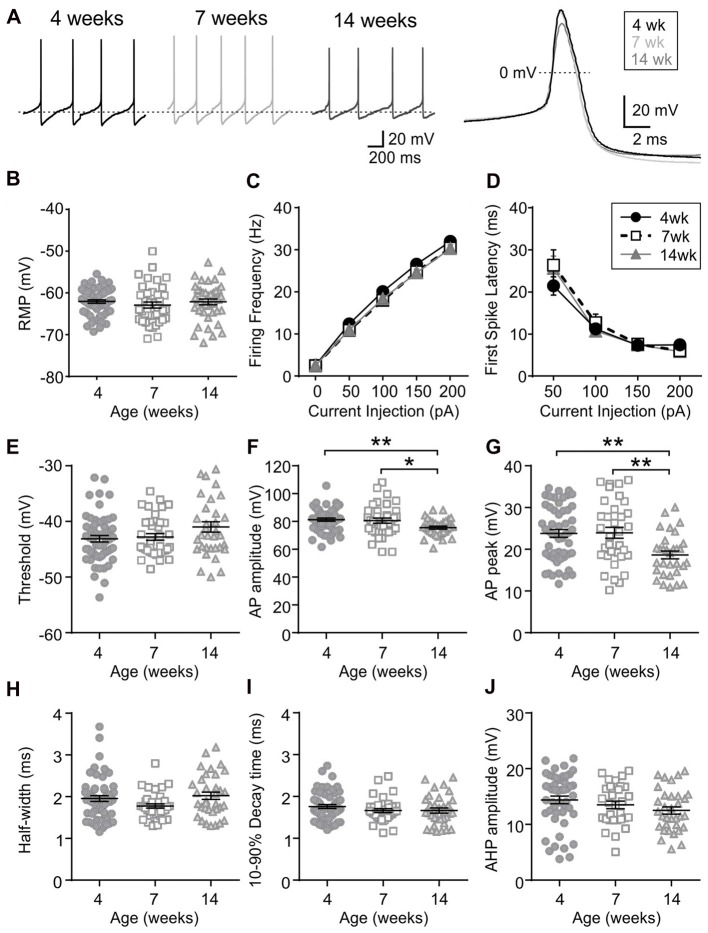
Excitability and AP waveform of orexin neurons after weaning. **(A)** Sample traces of spontaneous firing (left) and averaged AP waveform (right) recorded from orexin neurons of 4, 7 and 14-week old rats. Dotted line in the left panel is −59 mV. **(B)** RMP of orexin neurons from different age groups. **(C,D)** AP frequency **(C)** and first spike latency **(D)** of orexin neurons during positive current injections. **(E–J)** Various parameters of AP waveform as indicated on the Y-axis. *p < 0.05, **p < 0.01, one-way ANOVA with Holm-Sidak post-test.

**Figure 7:**
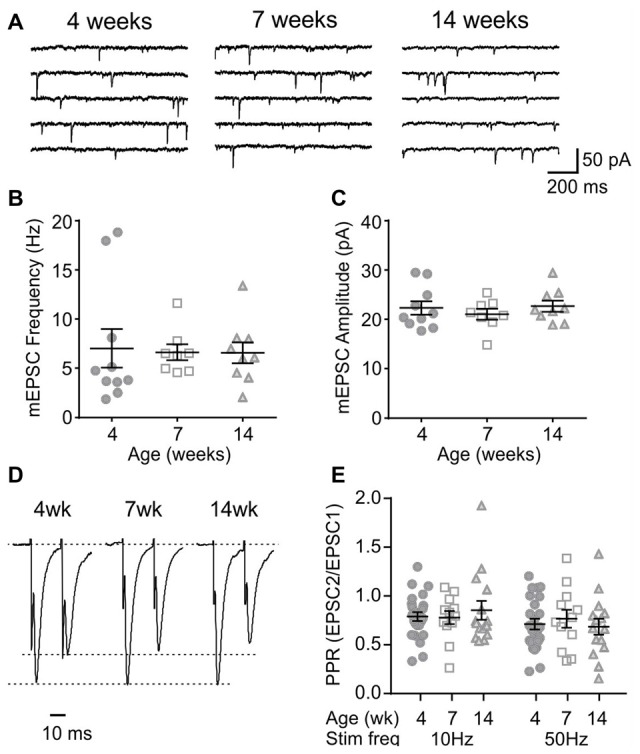
Basal properties of excitatory transmission to orexin neurons do not change after weaning. **(A)** Sample traces of mEPSCs in orexin neurons of 4, 7 and 14-week old rats. **(B,C)** The frequency **(B)** and amplitude **(C)** of mEPSCs in orexin neurons. **(D)** Paired EPSCs recorded from orexin neurons. Traces are scaled to the first EPSC. **(E)** Paired pulse ratio (PPR) at 10 Hz and 50 Hz stimulation.

Orexin neurons were spontaneously active (Figure 6A), as previously reported (Eggermann et al., 2003; Linehan et al., 2015). Among the age groups examined (4 weeks *n*/*N* = 58/18, 7 weeks *n*/*N* = 40/12, and 14 weeks *n*/*N* = 42/14), there was no age-dependent change in the RMP (*p* = 0.5400, one-way ANOVA), AP frequency (*p* = 0.0836, two-way RM ANOVA), first spike latency (*p* = 0.6239, two-way RM ANOVA) or threshold *p* = 0.0851, one-way ANOVA; Figures 6B–E). With respect to the AP waveform, there was a decrease in AP amplitude (*p* = 0.0087, one-way ANOVA; Figure 6F) and peak at 14 weeks (*p* = 0.0013, one-way ANOVA; Figure 6G); however, no changes were seen in other parameters such as the half-width (*p* = 0.0738), decay time (*p* = 0.2921) and AHP amplitude (*p* = 0.1469, one-way ANOVA; Figures 6H–J). Furthermore, no age-dependent change was observed in the frequency or amplitude of mEPSCs (4 weeks *n*/*N* = 10/4, 7 weeks *n*/*N* = 8/6 and 14 weeks *n*/*N* = 9/6; mEPSC frequency: *p* = 0.9699, mEPSC amplitude: *p* = 0.6352, one-way ANOVA; Figures 7A–C), or PPR at 10 Hz and 50 Hz (4 weeks *n*/*N* = 24/12, 7 weeks *n*/*N* = 12/8 and 14 weeks *n*/*N* = 15/5; *p* = 0.9548, two-way RM ANOVA; Figures 7D,E), indicating that there is no change in basal properties of excitatory synaptic transmission after weaning.

**Figure 8:**
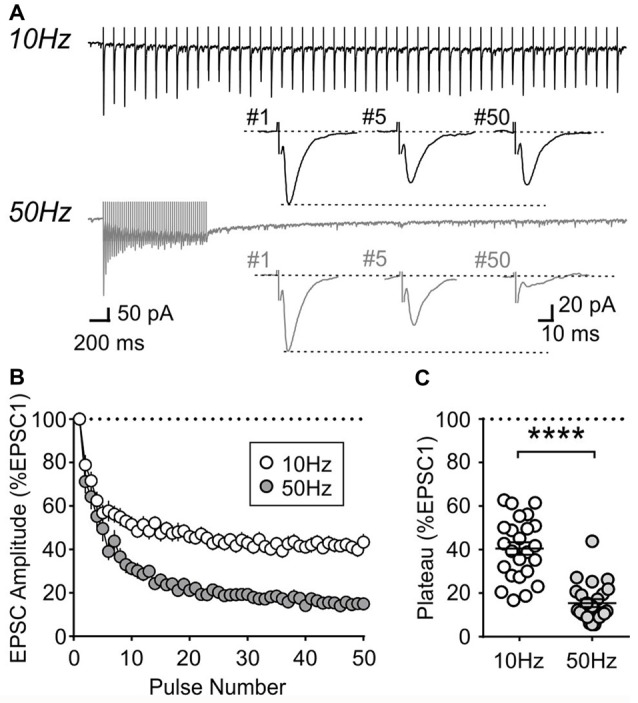
Activity-dependent short-term plasticity in orexin neurons of 4-week old rats. **(A)** Sample recordings of evoked EPSCs during 10 and 50 Hz train stimulation. Expanded traces below show the 1st, 5th and 50th EPSC of the train. **(B)** Normalized amplitude of EPSCs in response to10 and 50 Hz train stimulation. **(C)** Plateau of EPSC amplitude (average of last five EPSCs normalized to EPSC1) during 10 Hz and 50 Hz train stimulation. ****p < 0.0001, paired *t*-test.

**Figure 9:**
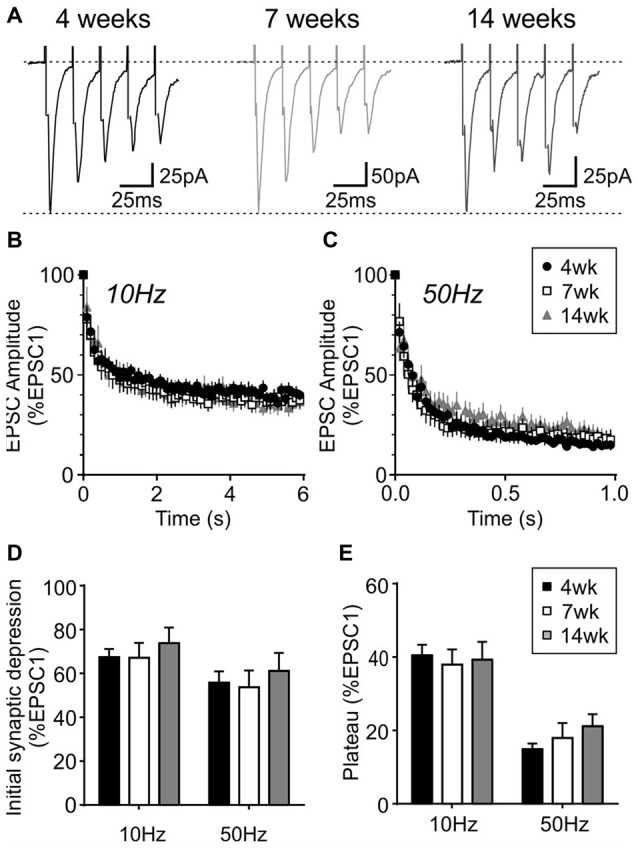
Activity-dependent short-term plasticity does not change in orexin neurons after weaning. **(A)** Sample traces of five EPSCs evoked at 50 Hz in orexin neurons from different age groups. **(B,C)** Normalized EPSC amplitude during 10 Hz **(B)** or 50 Hz train stimulation **(C)**. Note that the 4-week data is also shown in Figure 8B. **(D)** Synaptic depression at the beginning of 10 and 50 Hz trains (average of EPSC2–5, normalized to EPSC1). **(E)** Plateau at the end of 10 Hz and 50 Hz trains (average of EPSC26–30, normalized to EPSC1).

During repetitive activation, excitatory synapses to orexin neurons displayed synaptic plasticity that is distinct from that of MCH neurons. During 10 and 50 Hz train stimulation, these neurons showed robust short-term depression, which was greater in magnitude at 50 Hz, reaching a significantly lower plateau at the end of the train compared to that at 10 Hz (4 week *n*/*N* = 24/12, *p* < 0.0001, paired *t*-test; Figure 8). A similar short-term plasticity was seen at 7 and 14 weeks (7 weeks *n*/*N* = 12/8 and 14 weeks *n*/*N* = 15/5; initial synaptic depression *p* = 0.6655, plateau *p* = 0.8070, two-way RM ANOVA; Figure 9). Thus, electrophysiological properties of orexin neurons reach the adult level by 4 weeks of age in rats.

## Discussion

The present study demonstrates that during the post-weaning period in rats, MCH neurons experience an age-dependent decrease in excitability, which reaches the adult level by 7 weeks of age. In contrast, basic electrophysiological properties of orexin neurons are mostly stable from juvenile to adulthood (4–14 weeks of age). The decrease in the excitability of MCH neurons is characterized by hyperpolarization of RMP, depolarization of AP threshold and increased AHP amplitude, which most likely contribute to the decrease in AP frequency and prolonged first spike latency. A-type current could be developmentally regulated and may also affect the first spike latency (Falk et al., 2003). Moreover, it is important to note that the brain slices used in the present study were obtained during the light phase. This is typical of slice patch clamp studies on these neurons; however it is possible that the time of day has significant influence of their electrophysiological properties. These remain topics of future investigation.

These findings add to a previous study showing that the excitability of MCH neurons undergo age-dependent changes in mice. In mice, the RMP of MCH neurons plateaus around 4 weeks of age and remains stable into adulthood (Li and van den Pol, 2009), whereas we found that in rats, this plateau is reached by 7 weeks, suggesting species differences. During the postnatal period, GABAergic transmission is excitatory and contributes to developmental changes in the excitability of MCH neurons; however, GABA becomes inhibitory before weaning (Li and van den Pol, 2009), and our observed change in excitability persisted in the presence of GABA_A_ channel blocker. Thus, GABA is unlikely to underlie the excitability changes during the post-weaning period.

Excitatory transmission to MCH neurons also showed an age-dependent decrease. The amplitude of mEPSCs became smaller, suggesting a postsynaptic mechanism such as decreases in the surface expression or conductance of AMPA receptors (Pandey et al., 2015). mEPSC frequency also decreased between 4 weeks and 7 weeks, which could be secondary to reduced mEPSC amplitude; however, it also could be due to presynaptic mechanisms such as a decline in release probability or the number of active synapses (Fremeau et al., 2001; Horvath and Gao, 2005). Contrastingly, the PPR of MCH neurons decreased during the same period, suggesting a relative increase in release probability. This discrepancy may be explained by removal of low release probability synapses (Petralia et al., 1999; Hashimoto and Kano, 2003), mainly leaving higher release probability synapses intact. On the other hand, between 7 weeks and 14 weeks of age, mEPSC frequency does not show further change, while the PPR increases. This may indicate a reduced release probability of existing glutamatergic synapses.

This is the first study to report an activity-dependent short-term plasticity of excitatory synapses to MCH neurons. These synapses show a robust short-term facilitation, which is sustained if stimulated at 10 Hz. At 50 Hz, the facilitation is followed by synaptic depression, likely due to synaptic fatigue (Jackman and Regehr, 2017). Synaptic facilitation was transiently attenuated at 7 weeks of age. The reason for this is unclear; however, it may result from a relative increase in basal release probability. In contrast, excitatory synapses to orexin neurons show a strong short-term depression at both 10 and 50 Hz, with higher frequency resulting in greater depression as previously reported (Xia et al., 2009). Synaptic facilitation in MCH neurons would act as a high-pass filter, preferentially allowing high frequency signals to trigger postsynaptic firing (Fortune and Rose, 2001). On the other hand, synaptic depression in orexin neurons would act as a low-pass filter, which preferentially dampens high frequency signals while allowing sparse signals to trigger postsynaptic firing. These differences in synaptic responses and age dependence between MCH and orexin neurons can be expected to differentially affect the network activities within the lateral hypothalamus.

Notably, many changes in intrinsic excitability and synaptic transmission in MCH neurons occur between early (4 weeks old) and late adolescence (7 weeks old), whereas 4–5 weeks of age normally coincides with rodent growth spurt, onset of puberty and consolidation of adult sleep/wake rhythms (Spear, 2000; Hagenauer and Lee, 2013; Sengupta, 2013). MCH is known to play essential roles in energy homeostasis, body growth, and reproductive functions (Qu et al., 1996; Wu et al., 2009; Barson et al., 2013), and MCH knockout results in decreased bone mass, body length and body weight in adolescence and adulthood (Alon and Friedman, 2006; Mul et al., 2010). In addition, optogenetic activation of MCH neurons has been shown to induce REM and non-REM sleep (Jego et al., 2013; Blanco-Centurion et al., 2016), suggesting an important role of MCH in sleep/wake cycle. Thus, MCH maturation may also influence adolescent sleep patterns (Hagenauer and Lee, 2013). Therefore, electrophysiological maturation of MCH neurons during adolescence may broadly impact these physiological functions.

In contrast to MCH neurons, our results clearly indicate that the electrophysiological properties of neighboring orexin neurons are largely stable after weaning. Previous studies showed that developmental increases in orexin-immunopositive cell number and prepro-orexin mRNA expression reaches the adult levels between 2 weeks and 3 weeks of age in rats (Yamamoto et al., 2000; Sawai et al., 2010; Iwasa et al., 2015). Furthermore, electrophysiological properties of orexin neurons undergo developmental changes during the embryonic and postnatal period, including RMP, AP frequency and kinetics, and spontaneous synaptic activity (Ogawa et al., 2017). Taken together with our results, we conclude that orexin neurons are electrophysiologically mature before the adolescence period.

In summary, during the post-weaning period, MCH but not orexin neurons experience an age-dependent decrease in excitability. These changes may be critical in regulating energy balance associated with growth spurts and the sleep/wake cycle. In addition, our results suggest that age should be taken into consideration when designing experiments involving MCH neurons.

## Author Contributions

VL and MH contributed in the conception and design of the study, data analysis, data interpretation and writing of manuscript. VL performed the data acquisition. Both authors approve the version submitted and agree to be accountable for all aspects of the work.

## Conflict of Interest Statement

The authors declare that the research was conducted in the absence of any commercial or financial relationships that could be construed as a potential conflict of interest.
